# Neurological Sequelae in Adults After *E coli* O104: H4 Infection-Induced Hemolytic-Uremic Syndrome

**DOI:** 10.1097/MD.0000000000002337

**Published:** 2016-02-12

**Authors:** Ramona Schuppner, Justus Maehlmann, Meike Dirks, Hans Worthmann, Anita B. Tryc, Kajetan Sandorski, Elisabeth Bahlmann, Jan T. Kielstein, Anja M. Giesemann, Heinrich Lanfermann, Karin Weissenborn

**Affiliations:** From the Clinic for Neurology (RS, JM, MD, HW, ABT, KS, KW); Clinic for Nephrology (EB, JTK); and Institute for Interventional and Diagnostic Neuroradiology, Hannover Medical School, Hannover, Germany (AMG, HL).

## Abstract

In an outbreak of shiga toxin-producing *Escherichia coli* infections and associated hemolytic-uremic syndrome (STEC O104:H4) in Germany in the year 2011 neurological complications in adult patients occurred unexpectedly frequent, ranging between 48% and 100% in different patient groups. Few is known about the long-term effects of such complications and so we performed follow-up exams on 44 of the patients treated for STEC-HUS at Hannover Medical Scool in this observational study. Standardized follow-up exams including neurological and neuropsychological assessments, laboratory testing, magnetic resonance imaging (MRI), and EEG were carried out. Subgroups were examined 2 (n = 34), 7 (n = 22), and 19 (n = 23) months after disease onset. Additionally, at the 19-month follow-up, quality of life, sleep quality, and possible fatigue were assessed.

Nineteen months after disease onset 31 patients were reassessed, 22 of whom still suffered from symptoms such as fatigue, headache, and attention deficits. In the neuropsychological assessments only 39% of the patients performed normal, whereas 61% scored borderline pathological or lower. Upon reviewal, the follow-up data most prominently showed a secondary decline of cognitive function in about one-quarter of the patients. Outcome was not related to treatment or laboratory data in the acute phase of the disease nor length of hospitalization. Prognosis of STEC-HUS associated brain dysfunction in adults with regard to severity of symptoms is mostly good; some patients however still have not made a full recovery. Patients’ caretakers have to be aware of possible secondary decline of brain function as was observed in this study.

## INTRODUCTION

In May 2011, an unusual serotype of *Escherichia coli* (O104:H4) caused an outbreak of diarrhea-associated hemolytic uremic syndrome (HUS) in Germany. This strain combined the virulence potentials of 2 different pathotypes: EHEC (entero-hemorrhagic *E coli*) with Shiga Toxin 2 (Stx) production (also called Shiga toxin producing *E coli* or STEC) and EAEC (entero-aggregating *E coli*) with aggregative adherence to endothelial cells.^1^ The combination of the prophage encoding Stx and multiple resistance factors probably caused the spread of the outbreak as well as its severity.^2^ The Robert Koch Institute, the federal public health institute in Germany, registered 855 cases of confirmed STEC O104:H4 infection with HUS in addition to 2987 pure gastrointestinal infections. Twenty-two percent of the adult patients affected developed HUS^3^ of whom more than half showed involvement of the central nervous system (CNS).^4,5^ The majority (58%) even met the ICD-10 criteria for a mental disorder.^6^ At Hannover Medical School 48 patients with STEC-HUS were treated between May and July 2011, 47 of whom displayed neurological symptoms varying from slight headaches or trouble finding words, to severe alterations of consciousness, epileptic seizures, and need for mechanical ventilation.^5^ Although the majority of patients made rapid recoveries, there are still patients reporting impairment in their daily activities and cognitive skills.

In the present study, we performed standardized long-term follow-up examinations including clinical, neuropsychological, and neuroradiological assessments in adult STEC-HUS patients after infection with *E coli* O104:H4 serotype aiming to describe and classify the neurological sequelae.

## SUBJECTS AND METHODS

**FIGURE 1:**
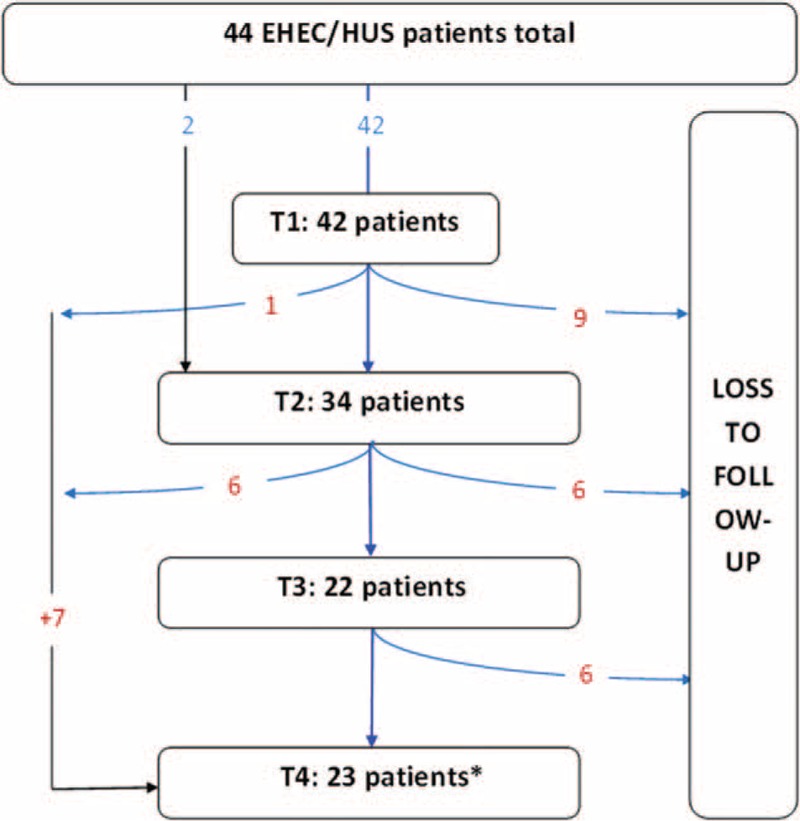
Diagram of loss to follow-up-SO: symptom onset, T1 acute phase of infection, T2-4 follow-up visits; PRES: posterior reversible encephalopathy syndrome ∗ patients, who were lost to follow-up but participated the questionnaire at T4.SO�=� symptom onset.

Of the 48 adult STEC O104:H4-HUS patients, who were treated at Hannover Medical School between May and July 2011, 44 had been regularly examined by a neurologist in the acute phase of the disease (T1) and/or after and thus were considered eligible for the study (Figure 1). About 2 months after symptom onset (Median 51 days, range 33–124 days) 34 of these patients underwent a first follow-up exam (T2), of whom 28 received an electroencephalogram (EEG). After 7 months (median 203 days, range 168–241) 22 patients attended a second follow-up (T3), magnetic resonance imaging (MRI) having been done on 20. Between 17 and 27 months after the symptom onset (median 576 days, range 524–820 days) 31 patients answered standardized self-report questionnaires, whereas 23 out of these attended the third follow-up (T4), EEGs having been performed in 20 and MRI in 13 (Figure 1, Table 1).

**TABLE 1:**
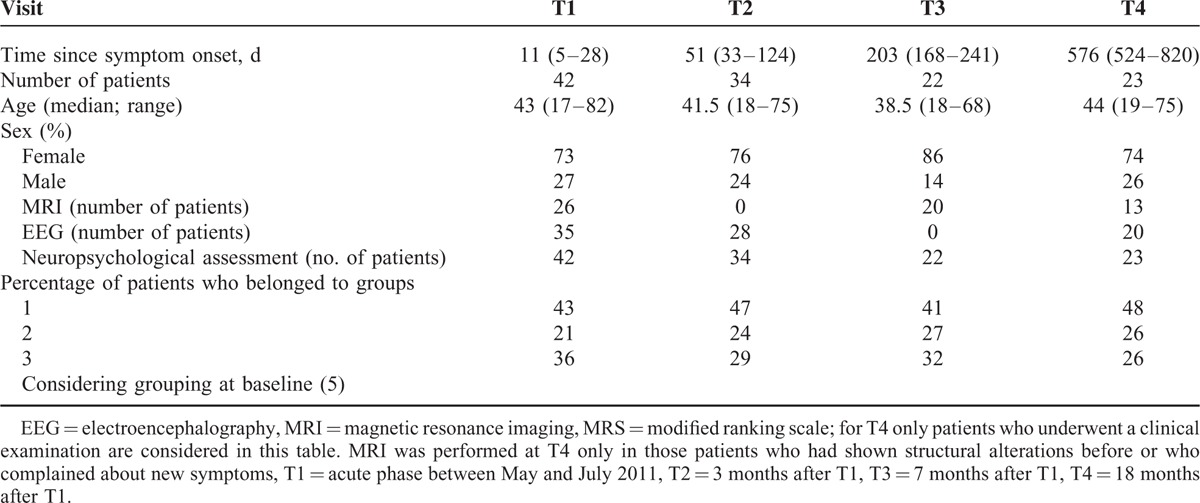
Distribution of Age and Sex in Study Subjects and Performed Examinations at Different Follow-Up Visits, MRI, EEG, MRS

Data collection and follow-up exams were approved by the local ethics committee (application numbers 1123–2011 and 1094–2011). Patients gave written informed consent for follow-ups.

The neuropsychological assessments at each follow-up comprised the tests “alertness,” “divided attention,” “orienting”, “attention shift,” and “working memory” of the test battery for the assessment of attention (TAP),^7^ the word-figure-memory test (WFMT),^8^ Luria's list of words,^9^ the Recurring Figures Test (RFT),^10^ and the Rey–Osterrieth–Complex–Figure Test^11^ in addition to the Mini Mental Status Examination (MMS). Individual test results were evaluated according to norm data and calculated into percentile ranks (PR). The 10th percentile was used as a cut-off between normal and pathological results (accordingly *z*-scores ≤−1.3); scores lower than that were considered clinically relevant.

The EEG was recorded according to the International 10–20-System using the EPAS Harmony System (Schwarzer, Germany). MRI of the brain was performed using a 1.5 Tesla Avanto or a 3 Tesla Verio (Siemens Medical Systems, Erlangen, Germany) and identical imaging parameters as in.^5^

At T4 patients were tested using the Epworth Sleepiness Scale (ESS),^12^ the Pittsburgh Sleep Quality Index (PSQI),^13^ the Fatigue Impact Scale (FIS),^14^ the Hospital Anxiety and Depression Scale (HADS),^15^ and the Short Form-36 questionnaire.^16^ All psychometric tests were completed in the German version and assessed using German norms.

Furthermore, the patients answered a questionnaire addressing current symptoms, job, and current medication. The ESS is a very short test to measure daytime sleepiness. Each item is rated on a 4-point scale; 10 points being the cut-off score for pathological daytime sleepiness (min 0, max 24). The PSQI is a subjective measure of sleep quality in the 4 weeks before completing the form. The patients answer 17 questions assesing sleep quality, latency, duration, efficiency, disturbance, use of sleeping medication, and daytime dysfunction. A score ≥ 5 can be considered suggestive of significant sleep disturbance (min 0, max 21). The FIS is a self-report scale to measure the impact of fatigue upon patients’ daily activities. It contains 40 items, each scored on a scale from 0 to 4. Sixty was chosen as a suggested cut-off for pathological fatigue (min 0, max 160) (14). The HADS serves for the detection of depression and anxiety in patients with internal and/or psychosomatic diseases. It is a 14 items scale, each scored from 0 to 3. A score ≥ 11 indicates anxiety or depression (min 0; max 21).

The short-form questionnaire (SF-36) is a survey with 36 questions to measure health-related quality of life (QoL). It provides scores for 8 health domains which can be summarized to a physical and a mental score which were calculated using normdata of the American population according to the questionnaire's manual. As a cut-off, a value outside the 2-fold standard deviation was chosen.

Follow-up visits T2 and T3 included analysis of serum creatinine, urea, glomerular filtration rate (GFR), C-reactive protein (CRP), white blood cell (WBC), and platelet count. At T1 S100-B and neuron-specific enolase (NSE) had been measured repetitiously as markers for the extent of brain damage. Peak levels were noted for analysing further correlation. All biochemical markers were determined using commercially available CE (Communautés Européennes)-certified reagents for clinical laboratory diagnostics.

To identify prognostic parameters, patients were subdivided into groups with good or poor outcome according to their SF-36 results and their answers in the questionnaire regarding current symptoms. Patients with remaining neuropsychiatric handycaps, reduced quality of life, and/or >2 abnormal test results (PR ≤ 10) and/or abnormal results in the assessment after completely normal performance in earlier follow-up visits were assigned to the poor outcome group (n = 15). Those with no or only slight subjective symptoms without relevance for daily activities and <3 abnormal results were sorted into the good outcome group (n = 16).

### Statistical Analysis

For comparison of outcome groups the Mann–Whitney–Wilcoxon test was used for continuous data and the chi-square and the Fisher exact test for categorical data, as appropriate. The Friedmann test was used for comparison of the different points in time. Analyses were performed using the SPSS software package 21 and 22. *P* < 0.05 was considered statistically significant.

## RESULTS

### Group Characteristics and Baseline Data

Data of the neurological presentation at T1 were decribed before.^5^ Of the 47 STEC-HUS patients with neurological symptoms seen at disease onset at Hannover Medical School, 42 underwent a standardized neurological assessment. Seven of these did not attend any of the follow-up visits: 1 patient died, another had been transferred to a rehabilitation clinic with a posterior reversible encephalopathy syndrome, and 3 refused participation. Two patients only answered the questionnaires per letter at T4 because they lived in quite a long distance to our clinic (see Figure 1).

At baseline (T1) patients were subdivided into 3 groups according to their worst neurological status during the hospital stay:^5^ Group 1 consisted of 18 patients (43%) who had presented with a normal MMS and normal Glasgow Coma Scale score (GCS) but suffered from slight clinical signs such as hyperreflexia, dysmetria, dysphasia, or slight headache. Nine patients (21%) who had been alert, but had achieved <28 points in the MMS had been assigned to Group 2, whereas Group 3 comprised 15 patients (36%) who had presented with alterations of consciousness during the acute phase. Those 2 patients who had not been examined by a neurologist at T1 were retrospectively assigned to Group 1 based on their anamnesis. These 3 groups were represented at the different follow-ups in equal distribution as at T1 (Table 1).

### Results of Neuropsychological Assessment, Laboratory Data, MRI, and EEG at T2-T4

Details about the number of patients included into the follow-up examinations are given in Table 1. Two months after disease onset (T2) 29% of the patients (10/34) achieved normal results (PR > 10) in all subtests of the neuropsychological assessment, 62% scored borderline or lower (PR ≤ 10) in 1 or 2 and 9% in >2 subtests. The patients reported slight neurological symptoms—mainly dysphasia, chronic fatigue, and headache—in 65%, whereas the neurological examination was normal in all patients. EEG was abnormal in 2 patients (7%), with general slowing in 1 and focal slowing in the other. Both had shown a abnormal EEG at T1 as well. The renal function was impaired in 35% (GFR level < 60 mL/min).

At T3 59% (13/22) of the patients achieved normal results in the neuropsychological assessment (PR > 10), 27% scored borderline or lower (PR ≤ 10) in 1 or 2 and 14% in >2 subtests. Neurological symptoms—mainly fatigue and concentration deficits—were reported by 64% of the patients. The impaired renal function (GFR < 60) was still detectable in 3 patients. White matter lesions, present in the acute phase on diffusion weighted MRI, were completely resolved. However, 11 out of 20 studies showed at least 1 new microangiopathic lesion compared to baseline. Two patients showed an increase of microangiopathic lesions of >8 each.

At T4 39% (9/23) of the patients achieved normal results in the neuropsychological assessment, 44% scored borderline or lower in 1 or 2 and 17% in >2 subtests. A total of 71% of the patients complained about slight neurological symptoms. Cognitive impairment and attention deficits were most frequently reported (37% and 43%). EEG was still abnormal in 1 patient with general slowing (theta waves). She also performed worse in the neuropsychological assessment at T4 than at T3. MRI showed an increase of microangiopathic lesions in only 2 cases, and no change in 9. Two patients who received an MRI at T4 for the first time showed no pathology. Table 2 summarizes the results of the neuropsychological assessment of 16 patients, who attended all follow-up visits. These 16 patients significantly improved over time with regard to their attention ability, whereas they showed a secondary decrease of their verbal learning ability and their visuo-constructional ability at T4. Memory retrieval improved over time in the Rey Complex Figure Test, whereas there was no change in the recognition of words or figures in the WFMT, and no change in the retrieval of words in the Luria List of Words test.

**TABLE 2:**
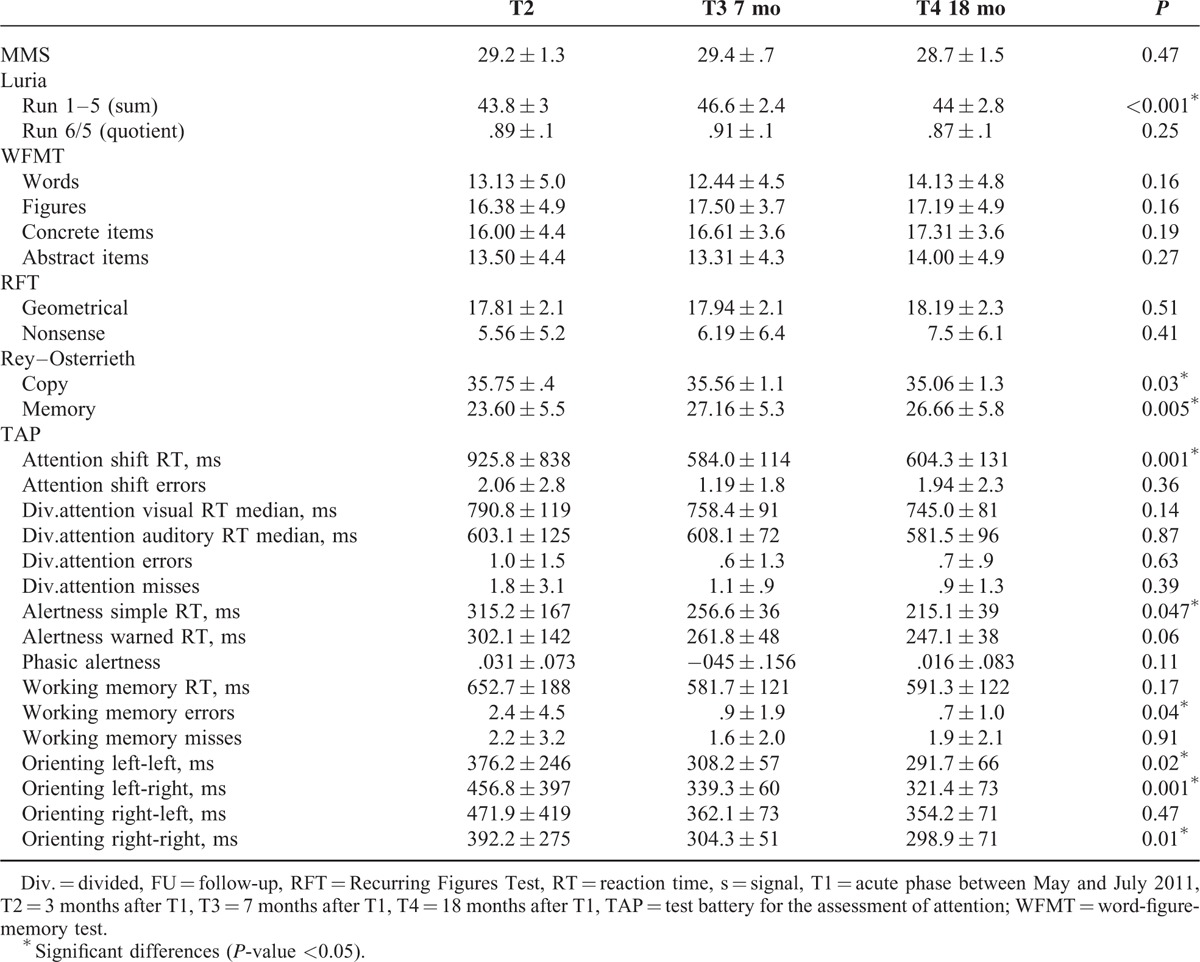
Results of the Neuropsychological Assessment of the 16 Patients Who Attended All 3 Follow-Up Visits, for the MMS, Luria’s List of words, WFMT; RFT and Rey-Osterrieth a Higher Score is Improvement, for the Subtests of the TAP-Battery a Lower Score is Improvement *P* Value, Using the Friedmann Test for Comparison of the 3 Different Points in Time, ^∗^Significant Differences (*P* Value < 0.05)

### Change of Cognitive Function From T2 to T4

**FIGURE 2:**
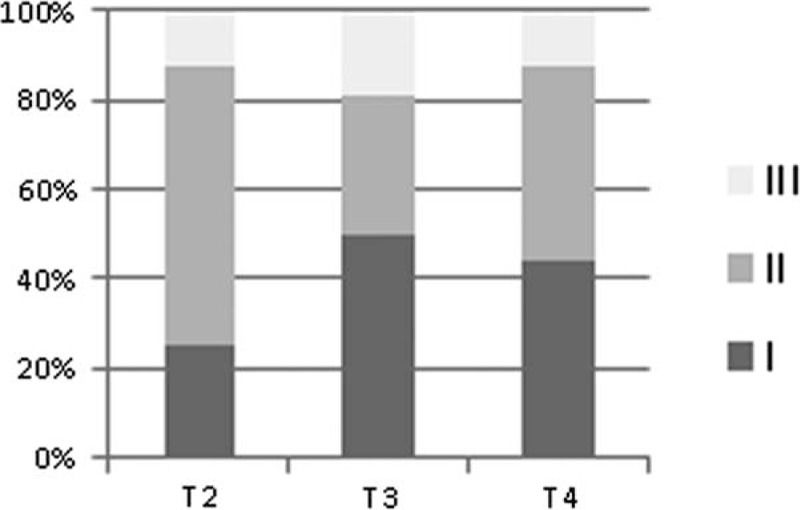
Results of the neuropsychological assessment for the 16 patients who attended all 3 follow-up visits, I normal result in all subtests (PR > 10), II 1–2 subtests with abnormal results (PR ≤10), III >2 subtests with abnormal results (PR ≤10). PR = percentile rank.

**FIGURE 3:**
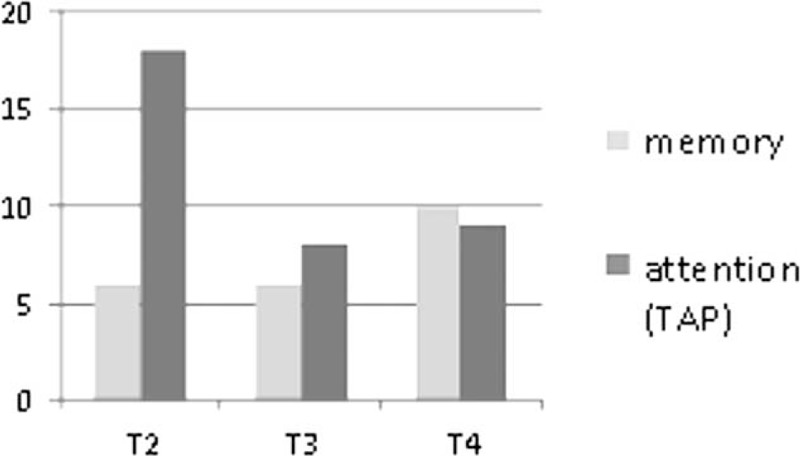
Number of abnormal test results, attributed to the main functions tested: “memory and attention” at the 3 follow-up examinations for the 16 patients.

**FIGURE 4:**
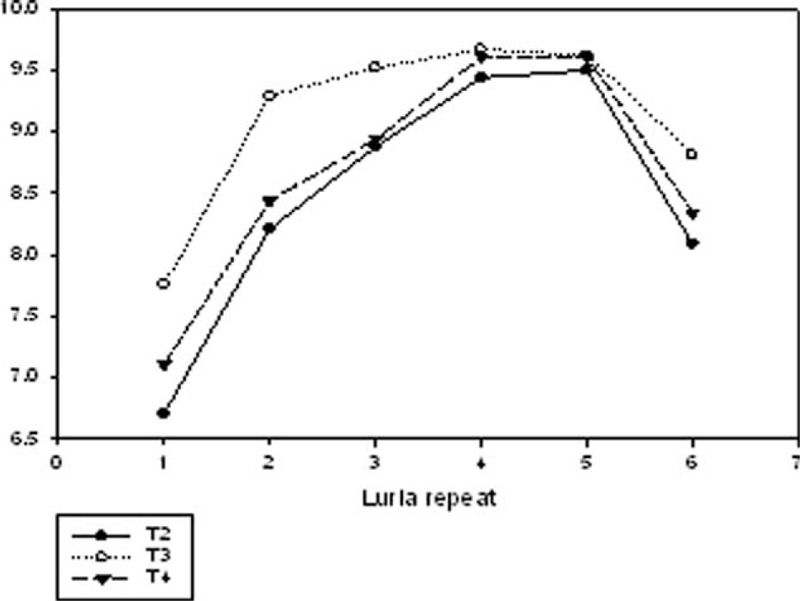
Recalled items of Luria’s list of words for run 1–6 at the 3 follow-up visits. Significant differences between the 3 points-in-time could be observed for Luria 1 (*P* = 0.013), Luria 2 (*P* = 0.009), and for the sum score (*P* < 0.001, not illustrated in the graph). Data are presented as mean.

In the follow-up examinations some of the patients reported new symptoms at T3 and T4, with memory decline and loss of visual function being the most frequently mentioned. At T4 the performance in the neuropsychological assessment declined compared to former examinations in 7 of the 23 patients tested. Figure 2 shows the percentage of normal, borderline, and abnormal test results in 16 patients who underwent all follow-up examinations. The mean of abnormal test results per patient declined from T2 to T3 (1.44–0.88) and increased from T3 to T4 (1.13) again in these 16 patients. The total number of abnormal test results increased in memory function tests and decreased in attention tests from T2 to T4 (Figure 3). Significant improvement was seen from T2 to T3 in the Rey–Osterrieth–Memory Test (*P* = 0.017) and in the attention shift test (*P* = 0.001). Between T2 and T4 significant improvement was seen in the simple reaction time in the alertness test (*P* = 0.026), the number of errors in the working memory test (*P* = 0.035) and 3 subtests of the orienting test (left–left *P* = 0.024, left–right *P* = 0.001, right–right *P* = 0.008). In Luriás List of Words (sum score), however, patients scored significantly worse at T4 compared to T3 (*P* = 0.002), whereas they had significantly improved from T2 to T3 (*P* = 0.001) (for subscores see Figure 4). In the MMS, which was the only test we could perform in the acute phase (MMS [T1] mean: 21.0 ± 11.2), the score significantly improved from T1 to T3 (*P* = 0.013).

### Results of Standardized Questionnaires (T4)

Patients complained at T4 about headache (23%), loss in physical fitness (30%), chronic fatigue (30%), sleeping disorders (23%), dysphasia (23%), gait disorder (30%), attention-deficit (43%), visual disturbances (27%), and cognitive impairment (37%). However, only 1 patient reported having lost her job due to the aftereffects of the disease. One of the 31 patients did not answer this questionnaire.

The ESS and PSQI scores suggested that 55% of the patients suffered from significant sleep disturbances (ESS score ≥ 10 and/or PSQI score ≥ 5) at T4 and 19% were affected by fatigue in their daily life (FIS > 60). One of the patients achieved an abnormal depression score in the HADS, and 1 an abnormal anxiety score (> 10); 2 of the 31 patients did not complete the HADS questionnaire. In the SF-36 1 patient scored abnormal in the physical sum score and 4 in the mental sum score (< 2 SD).

### Outcome Parameters

The poor (n = 15) and the good outcome group (n = 16), as defined above in subjects and methods, did not differ regarding age or sex nor with regard to other questionnaire results than the SF-36 (see Table 3). Furthermore there were no significant differences regarding laboratory parameters such as minimum platelet count, maximum white blood cell count, S100max, NSE at T1, minimum serum sodium at T1, GFR levels at T1, T2, or T3, as well as length of hospital stay and MMS at T1. Moreover, there were no differences regarding treatment at T1 (tested for immunoadsorption, plasma exchange, eculizumab). By defining the outcome groups only according to the patientś subjective impairment (at least 2 symptoms at T4 which affect daily activities), the poor outcome group (n = 12) was significantly older (*P* = 0.010) and more severely affected in the acute phase (*P* = 0.010).

**TABLE 3:**
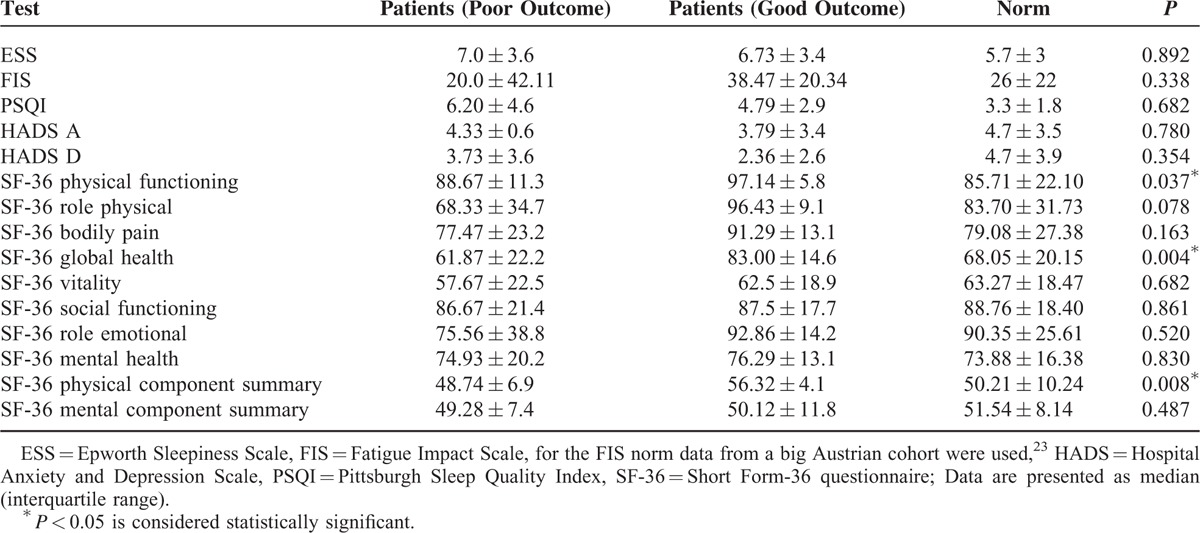
Results of the Questionnaires, n = 31, Except for the HADS (n = 29), ESS: Epworth Sleepiness Scale, FIS: Fatigue Impact Scale, for the FIS Norm Data From a Big Austrian Cohort Were Used (23), HADS: Hospital Anxiety and Depression Scale, PSQI: Pittsburgh Sleep Quality Index, SF 36: Short Form-36 Questionnaire; Data Are Presented as Median (Interquartile Range). *P* < 0.05 Is Considered Statistically Significant

## DISCUSSION

This study presents follow-up data from a cohort of adult patients from the German STEC O104:H4 induced HUS outbreak in 2011 2, 7, and 19 months after disease onset. Overall, the psychometric test results showed an improvement over time. However, more than half of the patients scored borderline or abnormal in the neuropsychological assessment 19 months after disease onset. Both the percentage of patients who complained about neuropsychiatric symptoms and the percentage of patients with abnormal or borderline neuropsychological test results increased at 19 months after an intermittent improvement at 7 months compared to 2 months after disease onset.

The main unexpected features of the 2011 outbreak were the high rate of adult patients (∼88%) and the frequent neurological involvement.^4,5^ Until now little is known about the long-term course of STEC-HUS in adults, but the significantly higher rate of neurological involvement in the acute phase compared to children (20–30% vs 48–100%)^4,5,17,18^ suggests to monitor neurological sequelae in addition to renal function also in the long term.

In children neurological sequelae of STEC-HUS are reported in only 4% of all patients, but in 50% of those with initial neurological complications after 4 to 7 years.^19^ Most frequent are hemiparesis, cortical blindness, and epilepsy.^20,21^ In contrast none of our adult patients showed seizures or clinical signs such as paresis or aphasia in the long-term follow-up. However, 27% of our patients reported visual disturbances similar to those previously observed in preschool children.^22,23^ One of our patients developed a bilateral anterior ischaemic optic neuropathy with persistent visual field defects and decrease of visual acuity to 70%.

The white matter lesions that had been observed especially in diffusion weighted magnetic resonance images of the brain in about half of the patients had resolved after 7 months (T3). In another cohort of this outbreak, MRI showed persisting lesions in 40% of the patients 37.1 ± 24.1 days after the infection.^24^ The discrepancy suggests a recovery between 2 and 7 months after HUS onset. But detailed data are missing. Several of our patients complained limitations in daily tasks such as leading a professional discussion, handling things simultaneously (“multitasking”), or doing mental calculations. These complaints are reflected by reduced performance in verbal learning and visuo-constructional ability at T4.

So far only a few studies addressed cognitive impairment after HUS. In 2 pediatric studies no significant cognitive impairment was detected 6 and 12 months after diarrhea associated HUS although a comprehensive neuropsychological test battery was applied.^21,25^ Recently early application of Eculizumab was suggested to improve the neurological outcome in children.^26^

A recent study that assessed 20 adult patients ∼3 months and 1 year after the acute disease, reported cognitive impairment in almost half of the patients 1 year after infection.^27^ Here again fatigue, psychomotor slowing, and concentration problems were reported frequently. Of note, the neuropsychological assessment was performed at 1 year only in those patients and in those tests where results below average had been observed in the first follow-up 10 to 30 weeks after disease onset. In our study the complete neuropsychological assessment was repeated at every follow-up. Thereby we were able to detect a secondary decline in performance after an initial improvement in about one-quarter of our patients. This 2-phasic course is a new aspect of STEC-HUS in adult patients and must be considered for the long-term care.

Secondary decline after initial improvement has been described several times for renal function in children with HUS,^18,19,28,29^ but the mechanism behind is still unknown—as it is for secondary cognitive decline.

Neurological symptoms in the acute phase of the disease are either due to Shiga-toxin induced neuronal damage or to antibody-related neuroinflammation. The latter hypothesis is supported by the delayed onset of neurological complications and their excellent response to immunoadsorption.^30^ A systemic inflammatory response to the infection may play a role as well. Serum IL-6, soluble tumor necrosis factor receptor 1 (sTNFR1), and tissue inhibitor of metalloproteinase-1 (TIMP-1) levels are elevated in HUS encephalopathy compared to HUS alone.^31^ Proinflammatory cytokines and especially TNF-alpha are known to induce neurodegeneration directly through signaling death pathway of TNF-α/p55 TNF receptor-1 in neurons.^32^

Stx consists of an enzymatic subunit A and 5 receptor-binding B subunits which bind to the glycolipid receptor globotriaosylceramide (Gb3) on the surface of endothelial cells and neurons, whereupon Stx enters the cell by endocytosis. Subunit A inactivates protein synthesis and induces cell death. By damaging endothelial cells, Stx impairs the blood–brain barrier function, thereby getting access also to brain cells.^33^ Animal experiments showed microglial activation and neuronal lesions with focal dendritic thickening and swelling in response to Stx.^32,34,35^

The hippocampus and the basal ganglia appear to be particularly vulnerable to Stx. Intravenous administration of sublethal doses of Stx in mice showed a correlation between neurological symptoms assessed by motor behavioral tests and the damage observed in the striatum and the hippocampus via transmission electron microscopy.^35^ Of note we were able to show microstructural alterations in the basal ganglia during the acute phase of STEC-HUS using quantitative MRI.^36^ In contrast the postmortem examination of the brain of 5 STEC-HUS patients from the 2011 outbreak did not reveal any endothelial or neuronal injury but just upregulation of the Stx receptor CD77/Gb3, a higher neuronal expression of interleukin 1ß and slight microglia activation.^37^ Thus, a possible mechanism of secondary brain damage after the acute phase of the disease remains elusive.

One limitation of our study is the small sample size, which precluded multivariate analyses. However, we were able to follow-up a very valuable subgroup of 16 patients by performing extensive examinations at all time-points, in order to understand the specific time course of the disease. The lack of baseline data is a further limitation of our study, but cannot be avoided due to the nature of the disease.

Future studies should address chronic microstructural alterations or ongoing microglial activation as possible causes of the 2-phasic course of cognitive dysfunction in adult STEC-HUS patients.

## References

[1] Bielaszewska M Mellmann A Zhang Characterisation of the *Escherichia coli* strain associated with an outbreak of haemolytic uremic syndrome in germany, 2011: a microbiological study. *Lancet Infect Dis* 2011; 11:671–676. 21703928 10.1016/S1473-3099(11)70165-7

[2] Rasko DA Webster DR Sahl JW Origins of the *E coli* strain causing an outbreak of hemolytic-uremic syndrome in germany. *N Engl J Med* 2011; 365:709–717. 21793740 10.1056/NEJMoa1106920 PMC3168948

[3] Frank C Werber D Cramer JP Epidemic profile of shiga-toxin-producing *Escherichia coli* O104:H4 outbreak in germany. *N Engl J Med* 2011; 365:1771–1780. 21696328 10.1056/NEJMoa1106483

[4] Magnus T Rother J Simova O The neurological syndrome in adults during the 2011 northern german *E coli* serotype O104:H4 outbreak. *Brain* 2012; 135 (Pt 6):1850–1859. 22539260 10.1093/brain/aws090

[5] Weissenborn K Donnerstag F Kielstein JT Neurologic manifestations of *E coli* infection-induced hemolytic-uremic syndrome in adults. *Neurology* 2012; 79:1466–1473. 22993286 10.1212/WNL.0b013e31826d5f26

[6] Kleimann A Toto S Eberlein CK Psychiatric symptoms in patients with shiga toxin-producing *E coli* O104:H4 induced haemolytic-uremic syndrome. *PLoS One* 2014; 9:e101839. 25007072 10.1371/journal.pone.0101839 PMC4090208

[7] Zimmermann P Fimm B Neuropsychologische testbatterie zur erfassung von aufmerksamkeitsdefiziten - rezidivierte fassung.

[8] Weissenborn K Ruckert N Brassel F A proposed modification of the wada test for presurgical assessment in temporal lobe epilepsy. *Neuroradiology* 1996; 38:422–429. 8837083 10.1007/BF00607265

[9] Christensen A Luriás neuropsychological investigation.

[10] Rixecker H Hartje W Kimura's recurring-figures-test: a normative study. *J Clin Psychol* 1980; 36:465–467. 7372815 10.1002/jclp.6120360213

[11] Osterreith P Le test de copie d́une figure complexe. *Arch Psychol* 1944; 30:206–356.

[12] Johns MW A new method for measuring daytime sleepiness: the epworth sleepiness scale. *Sleep* 1991; 14:540–545. 1798888 10.1093/sleep/14.6.540

[13] Buysse DJ Reynolds CF3rd Monk TH The pittsburgh sleep quality index: a new instrument for psychiatric practice and research. *Psychiatry Res* 1989; 28:193–213. 2748771 10.1016/0165-1781(89)90047-4

[14] Fisk JD Ritvo PG Ross L Measuring the functional impact of fatigue: initial validation of the fatigue impact scale. *Clin Infect Dis* 1994; 18 Suppl 1:S79–83. 8148458 10.1093/clinids/18.supplement_1.s79

[15] Zigmond AS Snaith RP The hospital anxiety and depression scale. *Acta Psychiatr Scand* 1983; 67:361–370. 6880820 10.1111/j.1600-0447.1983.tb09716.x

[16] Bullinger M Alonso J Apolone G Translating health status questionnaires and evaluating their quality: the IQOLA project approach. International quality of life assessment. *J Clin Epidemiol* 1998; 51:913–923. 9817108 10.1016/s0895-4356(98)00082-1

[17] Hahn JS Havens PL Higgins JJ Neurological complications of hemolytic-uremic syndrome. *J Child Neurol* 1989; 4:108–113. 2715605 10.1177/088307388900400206

[18] Siegler RL Spectrum of extrarenal involvement in postdiarrheal hemolytic-uremic syndrome. *J Pediatr* 1994; 125:511–518. 7931868 10.1016/s0022-3476(94)70001-x

[19] Rosales A Hofer J Zimmerhackl LB Need for long-term follow-up in enterohemorrhagic *Escherichia coli*-associated hemolytic uremic syndrome due to late-emerging sequelae. *Clin Infect Dis* 2012; 54:1413–1421. 22412065 10.1093/cid/cis196

[20] Bale JF Jr Brasher C Siegler RL CNS manifestations of the hemolytic-uremic syndrome. Relationship to metabolic alterations and prognosis. *Am J Dis Child* 1980; 134:869–872. 7416114 10.1001/archpedi.1980.02130210053014

[21] Schlieper A Orrbine E Wells GA Neuropsychological sequelae of haemolytic uremic syndrome. Investigators of the HUS cognitive study. *Arch Dis Child* 1999; 80:214–220. 10325699 10.1136/adc.80.3.214 PMC1717871

[22] Sheth KJ Swick HM Haworth N Neurological involvement in hemolytic-uremic syndrome. *Ann Neurol* 1986; 19:90–93. 3947042 10.1002/ana.410190120

[23] Eriksson KJ Boyd SG Tasker RC Acute neurology and neurophysiology of haemolytic-uremic syndrome. *Arch Dis Child* 2001; 84:434–435. 11316694 10.1136/adc.84.5.434 PMC1718775

[24] Lobel U Eckert B Simova O Cerebral magnetic resonance imaging findings in adults with haemolytic uremic syndrome following an infection with *Escherichia coli*, subtype O104:H4. *Clin Neuroradiol* 2014; 24:111–119. 23811994 10.1007/s00062-013-0231-0

[25] Gitiaux C Krug P Grevent D Brain magnetic resonance imaging pattern and outcome in children with haemolytic-uremic syndrome and neurological impairment treated with eculizumab. *Dev Med Child Neurol* 2013; 55:758–765. 23659643 10.1111/dmcn.12161

[26] Pape L1 Hartmann H Bange FC Eculizumab in typical hemolytic uremic syndrome (HUS) with neurological involvement. *Medicine (Baltimore)* 2015; 94:e1000. 26091445 10.1097/MD.0000000000001000 PMC4616562

[27] Simova O Weineck G Schuetze T Neuropsychological outcome after complicated shiga toxin-producing *Escherichia coli* infection. *PLoS One* 2014; 9:e103029. 25050708 10.1371/journal.pone.0103029 PMC4106865

[28] Small G Watson AR Evans JH Hemolytic uremic syndrome: defining the need for long-term follow-up. *Clin Nephrol* 1999; 52:352–356. 10604642

[29] Spizzirri FD Rahman RC Bibiloni N Childhood hemolytic uremic syndrome in argentina: long-term follow-up and prognostic features. *Pediatr Nephrol* 1997; 11:156–160. 9090653 10.1007/s004670050248

[30] Greinacher A Friesecke S Abel P Treatment of severe neurological deficits with IgG depletion through immunoadsorption in patients with *Escherichia coli* O104:H4-associated haemolytic uremic syndrome: a prospective trial. *Lancet* 2011; 378:1166–1173. 21890192 10.1016/S0140-6736(11)61253-1

[31] Shiraishi M Ichiyama T Matsushige T Soluble tumor necrosis factor receptor 1 and tissue inhibitor of metalloproteinase-1 in hemolytic uremic syndrome with encephalopathy. *J Neuroimmunol* 2008; 196:147–152. 18410971 10.1016/j.jneuroim.2008.02.012

[32] Takahashi K Funata N Ikuta F Neuronal apoptosis and inflammatory responses in the central nervous system of a rabbit treated with shiga toxin-2. *J Neuroinflammation* 2008; 5:11 2094-5–11. 18355415 10.1186/1742-2094-5-11 PMC2330034

[33] Ling H Boodhoo A Hazes B Structure of the shiga-like toxin I B-pentamer complexed with an analogue of its receptor Gb3. *Biochemistry* 1998; 37:1777–1788. 9485303 10.1021/bi971806n

[34] Goldstein J Loidl CF Creydt VP Intracerebroventricular administration of shiga toxin type 2 induces striatal neuronal death and glial alterations: an ultrastructural study. *Brain Res* 2007; 1161:106–115. 17610852 10.1016/j.brainres.2007.05.067

[35] Tironi-Farinati C Geoghegan PA Cangelosi A A translational murine model of sub-lethal intoxication with shiga toxin 2 reveals novel ultrastructural findings in the brain striatum. *PLoS One* 2013; 8:e55812. 23383285 10.1371/journal.pone.0055812 PMC3561315

[36] Weissenborn K Bultmann E Donnerstag F Quantitative MRI shows cerebral microstructural damage in hemolytic-uremic syndrome patients with severe neurological symptoms but no changes in conventional MRI. *Neuroradiology* 2013; 55:819–825. 23559401 10.1007/s00234-013-1176-3

[37] Hagel C Krasemann S Loffler J Upregulation of shiga toxin receptor CD77/Gb3 and interleukin-1beta expression in the brain of EHEC patients with hemolytic uremic syndrome and neurologic symptoms. *Brain Pathol* 2015; 25:146–156. 24989888 10.1111/bpa.12166 PMC8029455

